# Role of a Concentration Gradient in Malaria Drug Resistance Evolution: A Combined within- and between-Hosts Modelling Approach

**DOI:** 10.1038/s41598-020-63283-2

**Published:** 2020-04-10

**Authors:** Suwat Romphosri, Suttikiat Changruenngam, Thanat Chookajorn, Charin Modchang

**Affiliations:** 1 Biophysics Group, Department of Physics, Faculty of Science, Mahidol University, Bangkok, 10400 Thailand; 2 Genomics and Evolutionary Medicine Unit (GEM), Center of Excellence in Malaria Research, Faculty of Tropical Medicine, Mahidol University, Bangkok, Thailand; 3 Centre of Excellence in Mathematics, CHE, Bangkok, 10400 Thailand; 4 Thailand Center of Excellence in Physics, CHE, 328 Si Ayutthaya Road, Bangkok, 10400 Thailand

**Keywords:** Computational biophysics, Biological physics, Computational science

## Abstract

Resistance to antimalarial drugs is currently a growing public health problem, resulting in more cases with treatment failure. Although previous studies suggested that a concentration gradient facilitates the antibiotic resistance evolution in bacteria, no attempt has been made to investigate the roles of a concentration gradient in malaria drug resistance. Unlike the person-to-person mode of transmission of bacteria, the malaria parasites need to switch back and forth between the human and mosquito hosts to complete the life cycle and to spread the resistant alleles. Here we developed a stochastic combined within- and between-hosts evolutionary dynamics model specific to malaria parasites in order to investigate the influence of an antimalarial concentration gradient on the evolutionary dynamics of malaria drug resistance. Every stage of malaria development in both human and mosquito hosts are individually modelled using the tau-leaping algorithm. We found that the concentration gradient can accelerate antimalarial resistance evolution. The gain in resistance evolution was improved by the increase in the parasite mutation rate and the mosquito biting rate. In addition, even though the rate of resistance evolution is not sensitive to the changes in parasite reduction ratios (PRRs) of antimalarial drugs, the probability of finding the antimalarial drug resistant parasites decreases when the PRR increases.

## Introduction

Malaria is a vector-borne disease caused by the parasitic protozoa in the *Plasmodium* species. There are five species of the *Plasmodium* protozoa, namely, *P. falciparum*, *P. vivax*, *P. ovale*, *P. malariae*, and *P. knowlesi* that can infect humans via the bite of infected *Anopheles* mosquitoes^1^. In 2017, the World Health Organisation (WHO) reported 219 million cases with 435,000 deaths worldwide^2,3^. Malaria is found in many tropical regions, and malaria was considered to be endemic in 91 countries in 2016^4^. Malaria treatment relies only on the administration of antimalarial drug regimens, and the first-line treatment for *P. falciparum* as recommended by WHO is artemisinin combination therapies (ACT), which are to date the most effective antimalarial drugs for uncomplicated *P. falciparum* malaria^5^. ACTs are the combination of artemisinin derivatives and partner drugs. Artemisinin affects the asexual blood stage and the early sexual stage of the parasite^6^, whereas a partner drug clears residual parasites from an artemisinin pulse. ACTs have been used to treat malaria since 1994 and are currently the most widely used malaria treatment regimen.

Nevertheless, resistance to antimalarial drugs is posing a growing public health threat^7,8^. Antimalarial drug resistance gives the parasite an opportunity to survive under antimalarial pressure. When resistance emerges, it prolongs the illness and delays the clearance time, resulting in treatment failure. This can also increase the mortality, morbidity, and transmissivity rates of the disease. Among the five species of *Plasmodium*, *Plasmodium falciparum* poses the most burden, especially in African countries, and is responsible for most malaria deaths^2^. Resistance to chloroquine, sulfadoxine and pyrimethamine are widespread, while delayed clearance following artemisinin treatment was reported in five countries in the Great Mekong subregion (Cambodia, Laos, Myanmar, Thailand, and Viet Nam). Moreover, the cases with reduced artemisinin effectiveness were first reported in 2004 near the border between Thailand and Cambodia^9^.

Many factors are likely to accelerate drug resistance evolution in general. One key factor is a drug concentration gradient which is known to promote the evolution of drug resistance in bacteria^10–13^. It has also been shown that antibiotic concentration gradients can arise in both a single-host level (e.g., different organs can absorb different amounts of antibiotics resulting in a concentration gradient between organs within a patient), as well as in a population level (e.g., bacteria can migrate between treated and untreated patients and, therefore, experience different drug concentrations)^14–16^. Antibiotic resistance, therefore, naturally evolves in environments with antibiotic concentration gradients.

Many experimental studies have investigated the effects of the concentration gradient in the drug resistance evolution of bacteria^10,11^. For instance, in 2011, the first experimental study of antibiotic resistance evolution in the heterogeneous environment was conducted using a microfluidic device^10^. The experiment revealed an important role of a concentration gradient in the resistance evolution of bacteria. Under the drug concentration gradient, the resistance to ciprofloxacin was found within 10 hours, as opposed to 25 hours with no emergence of drug resistance in the homogeneous environment^10^. This demonstrated that spatial heterogeneity can accelerate the drug resistance evolution in bacteria. In addition to experimental studies, there are also theoretical works investigating the roles of spatial heterogeneity in antibiotic resistance evolution^12,13^. For example, in 2012, a group of researchers introduced a staircase model for investigating the role of spatial heterogeneity in the evolution of antibiotic resistance^13^. In this model, a one-dimensional environment was divided into many small isolated compartments in which bacterial migration occurred between these compartments. The drug concentration in each compartment increased from left to right. Bacteria inhabiting each compartment proliferated (grew), migrated, mutated, and died. The results of the study showed that concentration gradients provide a mode of adaptation that is impossible to observe in uniform environments. The analytical and numerical results of this study showed that drug concentration gradients can promote antibiotic resistance.

Similar to bacteria, it has also been suggested that malaria parasites may often experience heterogeneous distribution of antimalarial drugs. This heterogeneity arises due to differences in health infrastructure and drug accessibility among human populations^17^. In addition, the circulation of fake and low-quality drugs in some groups of patients may also produce antimalarial concentration gradients among populations^18,19^. However, although the experimental and the theoretical works suggested that drug concentration gradients facilitate the antibiotic resistance evolution of bacteria^10–13^, to the best of our knowledge, no attempt has been made to investigate the roles of an antimalarial drug concentration gradient on the resistance evolution of malaria parasites. Unlike the spreading of antibiotic resistance in which the resistant bacteria can transmit directly from one person to another, the transmission of antimalarial drug resistance is much more complicated; the resistant parasites need to migrate back and forth between the human and mosquito hosts. The life cycle of malaria parasites is more complicated, as it requires both human and mosquito hosts to be completed.

In general, mathematical modelling and computer simulation can be used either as predictive tools or as a means of understanding fundamental physical and biological processes. They enable the prediction of outcomes that would not be possible to investigate in the laboratory and in the real world^20^. Evolutionary dynamics of antimalarial drug resistance also falls into this category. Unlike bacteria, to complete the whole life cycle, malaria parasites need to live in both mosquito and human hosts. This prohibits researchers from conducting experiments on the evolutionary transmission of malaria parasites in the laboratory setting.

In this work, we developed a combined within- and between-host evolutionary dynamics model of malaria parasites to investigate the roles of an antimalarial drug concentration gradient on the evolutionary dynamics of malaria parasites. In our model, all stages of malaria parasites in both human and mosquito hosts are explicitly modelled using a stochastic approach^21^. Parameter values in this model are based on available experimental and clinical data. A sensitivity analysis of certain model parameters that could affect the evolutionary dynamics of antimalarial drug resistance was also conducted.

## Methods

### The within-host parasite dynamics model

**Figure 1:**
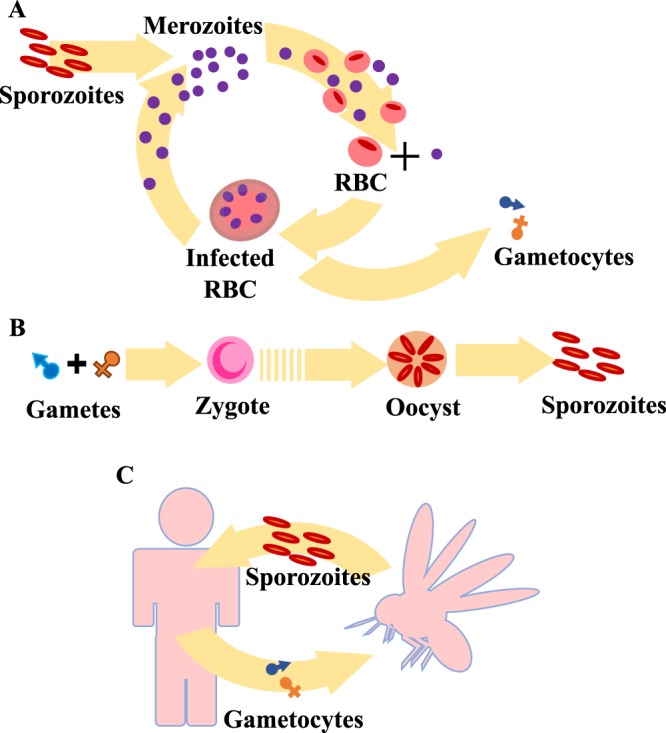
The parasite dynamics model. (**A**) A schematic of the malaria parasite dynamics in the human host. After 10 sporozoites are injected into the host body, they travel to the host liver and produce new offspring (merozoites). Each sporozoite produces merozoites at rate of 30,000 merozoites per 10 days. Each released merozoite invades a red blood cell at the rate of 1 − *N*/*K* and then begins the 48-hour reproduction cycle. In addition to merozoites, gametocytes may be produced from IRBCs at the ratio of 0.1. (**B**) A schematic of the malaria parasite dynamics in the mosquito host. A male and a female gamete combine together to form a zygote. This zygote develops into an oocyst that can then produce sporozoites at rate of 10,000 sporozoites per 10 days. (**C**) A schematic of the between-host transmission dynamics of malaria parasites.

This section describes the proposed computational population dynamics model of malaria parasites in human and mosquito hosts. The complete parasite life cycle in human and mosquito hosts is separately simulated using the *τ*-leaping algorithm^21^. The model simulation begins with 10 inoculated sporozoites in a human body. Each sporozoite infects the human liver and produces merozoites (the blood stage parasites) at the rate of 30,000 merozoites per 10 days^22,23^. The merozoites are then released to the bloodstream and invade the red blood cells at an invasion rate

1 $$r=\{\begin{array}{cc}1-N/K, & N < K\\ \,0, & N\ge K{\prime} \end{array}$$

where *N* is the number of infected red blood cells (IRBCs) in the human body and *K* is the parasite carrying capacity of the human host^22^. 1 − *N/K* is a logistic growth term representing the limitation of the growth rate caused by, for example, food and space limitations. The merozoites are produced from an IRBC at the rate of 10 merozoites per 2 days^24,25^. Occasionally, some sexual gametocytes are produced instead of asexual merozoites. The ratio of the number of gametocytes to the number of merozoites produced from an IRBC ranges from 0.001 to 0.40^26^. In our simulations, unless stated otherwise, this ratio is fixed at 0.1. The gametocytes are produced with the sex ratio of male to female gametocytes of 1:3.6^27^. The gametocytes can circulate in the human body for an average of 6.4 days^26^ and may be carried by a mosquito via biting at a rate of 0.344 per day^28^. The within-host model of malaria parasite dynamics in a human body is summarised in Fig. 1A.

In the mosquito host (Fig. 1B), male and female gametocytes both develop into mature gametes^24^. A male and a female gamete mates to form a zygote. This mobile zygote or ookinete then develops at the midgut in the form of oocyst for 7 days^29^. When an oocyst reaches the mature stage, it produces approximately 10,000 sporozoites^30^. The sporozoites within the mosquito saliva can be transferred to a human host via a mosquito bite. The parameters and their values used in this model are summarised in Table 1.

**Table 1:** A summary of the parameters and their values used in the within-host population dynamics model.

Definitions	Values	Refs.
Number of inoculated sporozoites via a mosquito bite	10 sporozoites	^22^
Production rate of merozoites from a sporozoite	30,000 merozoites per 10 days	^22,23,34^
Production rate of merozoites from an IRBC	10 merozoites per 2 days	^24,25^
Production rate of gametocytes from an IRBC	10 gametocytes per 10 days	^46^
Gametocyte to merozoite ratio	0.1	^26^
Average gametocyte circulation time	6.4 days	^26^
Parasite carrying capacity	10^12^ per host	^22^
Gametocyte sex ratio (male:female)	1:3.6	^27^
Parasite reduction ratio of artemisinin monotherapy	10^3^	^47^
Number of male gametes produced from a male gametocyte	8 gametes	^24^
Number of female gametes produced from a female gametocyte	1 gamete	^24^
Time from zygote to oocyst	7 days	^29^
Number of sporozoites produced from a mature oocyst	10,000 sporozoites per 10 days	^30^

### The between-host transmission dynamics model

The within-host parasite dynamics model proposed in the previous section can only separately describe the proliferation of the malaria parasites in an infected human and an infected mosquito host. However, to simulate the whole life cycle of malaria parasites within both human and mosquito hosts, the between-host transmission dynamics of the parasites are needed. The between-host transmission dynamics involve two separate events, namely, the transmission of gametocytes from an infectious human to a susceptible mosquito, and the transmission of sporozoites from an infectious mosquito to a susceptible human (Fig. 1C). These two events can separately occur through a mosquito bite and are governed by two parameters, namely, a biting rate, defined as the number of mosquito bites that each human individual receives in a day, and an infection probability describing a chance for a mosquito or a human to obtain an infection through a bite.

When a susceptible mosquito bites an infectious human, the mosquito can obtain an infection with an infection probability that is proportional to the density of the gametocytes within the infectious human at the time of biting^31^ (see Fig. S1 and the Supplementary Methods). If the bite leads to infection, gametocytes will be transferred to the mosquito. The number of gametocytes that will be transferred to the mosquito is proportional to the gametocyte density in the human body at the time of the biting. We assume that a mosquito takes 1 µL of blood during a blood meal. Based on a previous study^29^, the number of oocysts in a mosquito after blood feeding ranges from 1 to 200 (median 7.28) and the number of gametocytes per bite can range from 2 to 300; however, to reduce the simulation time, the maximum number of gametocytes per bite is limited to 20 gametocytes per bite. Limiting the maximum number of gametocytes per blood meal to 20 does not significantly affect the evolutionary dynamics of parasites because only one pair of male and female gametocytes is enough to infect a mosquito (see the analysis in the Sensitivity analysis section). While the probability of transmission of gametocytes from an infectious human to a susceptible mosquito depends on the density of the gametocytes within a target host^31^, the transmission of the sporozoites from an infectious mosquito to a susceptible human is assumed to definitely occur. When an infectious mosquito bites a human, 10 sporozoites are transferred into the human body^22^. In our model, the biting rate is fixed at 0.34 per human per day^28^.

### The spatial heterogeneity and evolutionary dynamics

To investigate the roles of the spatial heterogeneity in the antimalarial drug resistance dynamics, following the method presented in^13^, we consider a spatial environment that is divided into several relatively isolated compartments. Both the human and mosquito populations are also divided into subpopulations living in different compartments; hence, each compartment represents a co-living area of human and mosquito hosts (Fig. S2 in the Supplementary Material). All of the compartments are aligned in one-dimensional space.

The encounter rate between a human host living in compartment *i* and a mosquito inhabiting compartment *j* depends on the distance between compartments *i* and *j*. We assume that mosquitoes only stay in their home compartments while individual humans can travel to the other compartments. The encounter rate between a human individual and a mosquito can therefore be computed using a human mobility model. Following the human mobility model presented in ref. ^32^, the probability density that an individual living in compartment *i* will travel to compartment *j* (*P*_*m*_) is given by

2 $${P}_{m}({\Delta }{r}_{ij})={({\Delta }{r}_{ij}+{\Delta }{r}_{0})}^{-\omega }exp(\,-{\Delta }{r}_{ij}/\kappa ),$$

where ${\Delta }{{r}}_{{ij}}=|j-i|d\,$ is the distance between compartment *i* and *j*, $\omega =1.75$, $\kappa =80$, $\Delta {r}_{0}=1.5\,km$, and *d* = 1 km is the width of each compartment. Therefore, in our model, each human individual living in compartment *i* can be bitten by a mosquito inhabiting in any compartment *j* with a probability that is proportional to ${P}_{m}(\Delta {r}_{ij})$. It is worth noting that in this study, we did not explicitly simulate human movement between compartments, but instead, we used the travel probability density (Eq. 2) to estimate how likely a human individual will be bitten by a mosquito living in each compartment. By combining the travel probability density ${P}_{m}(\Delta {r}_{ij})$, the biting rate, and the mosquito infection probability ${P}_{Inf}(n)$, the parasites can migrate throughout the entire environment.

**Figure 2:**
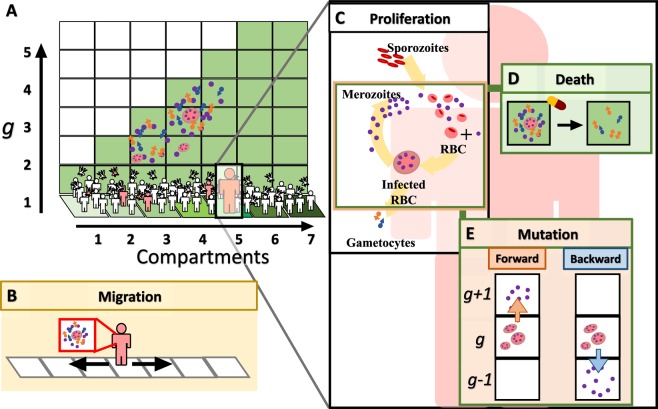
The evolutionary model of antimalarial drug resistance. (**A**) We consider an environment divided into several compartments (plotted horizontally) in which malaria parasites can (**B**) migrate through human travel and a mosquito bite, (**C**) proliferate (grow), (**D**) mutate, and (**E**) die due to drug treatments. The malarial genotypes are characterised by an integer *g*, plotted vertically in (**A**), representing antimalarial drug resistance levels. A mutation increases or decreases g by one. The concentration of an antimalarial drug, represented by the green shaded areas, increases from left to right. The parasites living in compartment i will be killed by a drug if they have *g* ≤ *i*.

Figure 2 illustrates the evolutionary model of antimalarial drug resistance in a heterogeneous environment. Following the method presented in^13^, we consider an environment that is divided into a series of relatively isolated compartments (Fig. 2A). Each compartment is associated with a fixed antimalarial drug concentration that linearly increases from left to right. The genotype of each parasite is characterised by a positive integer *g* plotted vertically. This integer *g* indicates a drug concentration level that can kill the parasites. For example, the parasites with *g* = 2 can be killed by a drug if they live in a compartment that is associated with the drug concentration level ≥ 2, that is, they can be killed if they live in a compartment *i* ≥ 2. Importantly, parasites with a larger *g* are more resistant and cannot be killed at a lower drug concentration level.

Each parasite is capable of proliferating, migrating via human travel and mosquito bites, getting killed by an antimalarial drug, and accumulating mutations in a human host. Although a mutation of the parasites may occur at any stage of their life cycle, in our model we assumed that the mutations occur mainly during the reproduction cycle of merozoites. This is reasonable because the number of parasites in the blood stage is highest; hence, it is more likely that the mutations will occur during this reproduction cycle. A forward mutation occurring at a rate of *μ*_*f*_ increases *g* by one, whereas a backward mutation does the opposite by decreasing *g* by one at a rate of *μ*_*b*_. Gametocytes with a genotype *g* can be produced from a merozoite with the same genotype *g*. On the other hand, in an infected mosquito, when a male gamete with a genotype *g*_*m*_ mates with a female gamete with a genotype *g*_*f*_, we assume that a zygote with either genotype *g*_*m*_ or *g*_*f*_ will be produced with equal chance. We have also checked that in all of our model realisations there is no gamete recombination in which *g*_*m*_ and *g*_*f*_ differ more than one. Parasite proliferation is simulated using the within-host parasite dynamics model (Fig. 1).

In addition, the population dynamics of parasites does not only vary from one compartment to another, but also depends on the disease states of each individual. We classify the human hosts into four categories, namely, susceptible, exposed, infectious, and recovered, based on the number of parasites within their bodies. When an infectious mosquito bites a susceptible human, parasites are transmitted into the human. After being infected, the susceptible human progresses to the exposed class. Humans in this class have already acquired the infection, but the numbers of parasites within their bodies are not high enough to be noticed. Therefore, the parasites within these exposed people will not encounter any antimalarial drug. In contrast, the number of parasites within infectious individuals is high enough to be noticed. Based on the study in^33^, approximately 82.9% of 76 febrile patients had a number of parasites greater than 10^9^. We therefore assume that the infected individuals with a number of parasites more than 10^9^ will receive an antimalarial drug treatment, and their state will change to the infectious state. After infectious individuals receive the drug treatment and all of the parasites in their bodies are cleared out, they progress to the recovered state. We assume that the recovered individuals gain the ability to suppress parasites. The recovered individuals may be re-infected through a bite from an infectious mosquito, but they will not develop any symptoms and will not seek treatment. The number of parasites within the re-infected recovered individuals will be suppressed below the detection limit of 10^8^ ^22^. The default values of the model parameters are summarised in Table 2. The combined within- and between-hosts evolutionary dynamics model was simulated using the *τ*-leaping algorithm^21^ with a time step (*τ*) of 0.1 days. All of the information related to each malaria parasite, human individual, and mosquito (e.g., parasite stage, human and mosquito infection stage, compartmental location *i*, genotypic index *g*) was recorded and updated at each time step *τ*.

**Table 2:** A summary of the default values of the model parameters.

Definitions	Values	Refs.
Number of human population in each compartment	100	[Assumed]
Number of mosquito population in each compartment	300	[Assumed]
Total number of compartments	10	[Assumed]
Total number of *g*	10	[Assumed]
Forward mutation rate	10^−7^ per generation	[Assumed]
Backward mutation rate	10^−4^ per generation	[Assumed]
Parasite reduction ratio	10^3^	^47^
Number of parasites that leads patients to receive treatments	10^9^	^33^
Maximum number of parasites within recovered individuals (below the detection limit)	10^8^	^22^
Biting rate	0.344 per person per day	^28^

## Results

### Parasite dynamics within the human and mosquito hosts

**Figure 3:**
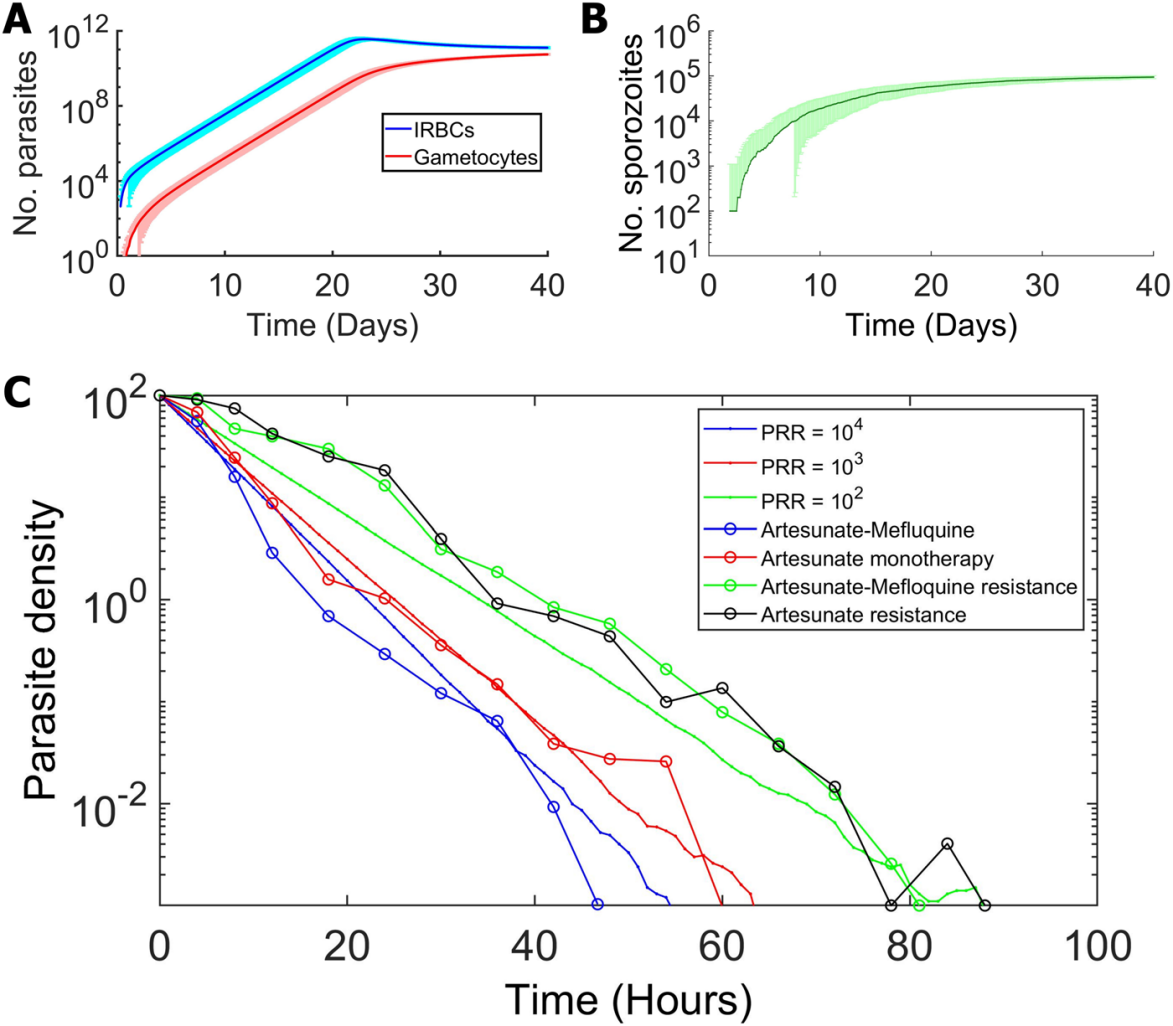
Population dynamics of parasites within an infected human and an infected mosquito. (**A**) The number of parasites in an infected human host as a function of time after the infection of the human host (the time since the susceptible human got the infection from the infectious mosquito). The blue and red curves show the number of infected red blood cells and gametocytes with their standard deviations, respectively. (**B**) The number of sporozoites in an infected mosquito as a function of time since the infection of the mosquito (the time since the susceptible mosquito got the infection from the infectious human) with the standard deviation. (**C**) Simulation results showing the parasite dynamics in the human host under antimalarial drug treatment. The parasite density represents the percentage of the number of parasites compared to that at the treatment starting time. The open circle lines show the experimental data (adapted from^35^), and the dashed lines show the simulation results.

Figure 3 shows the separated dynamics of parasites within an infected human host and a mosquito. In a human host, after being infected by a bite from an infectious mosquito, 10 sporozoites were initially transferred into the host body. Without any interference, the number of parasites increases rapidly and reaches the detection limit (10^8^ parasites) in 2 weeks, which approximately equals the time required for the liver schizont to release the first wave of merozoites^22,34^. After this point, the number of parasites continues to increase and approaches maximum of 10^12^ parasites within 3 weeks (Fig. 3A). In a mosquito vector, after being infected by biting an infectious human individual, gametocytes were transferred into the mosquito body. The number of sporozoites in the infected mosquito subsequently increases rapidly after the infection (Fig. 3B).

We then incorporated the effects of an antimalarial drug into our within-host parasite dynamics model and compared our simulation results with the available experimental data reported in^35^. Usually, malaria symptoms can be observed in 2 weeks after an infective mosquito bite^36^. Based on the study in^33^, 17.1% of 76 febrile patients had a number of parasites less than 10^9^. Therefore, in our model, if the number of parasites reaches 10^9^, the drug treatment will be administered. As shown in Fig. 3C, our simulation results agreed with the observational data. The parasite reduction ratios (PRRs), defined as the ratio of parasitaemia numbers at the initial treatment time to the number at 48 hours, of artesunate monotherapy and artesunate-mefloquine combination therapy were approximately 10^3^ and 10^4^, respectively. In addition, as resistance to antimalarial drugs results in delayed parasite clearance time and the reduction in PRRs, our simulation results indicate that both PRRs of artesunate monotherapy and artesunate-mefloquine combination therapy of resistant parasites drop below 10^2^.

### Roles of a concentration gradient on the evolutionary dynamics

**Figure 4:**
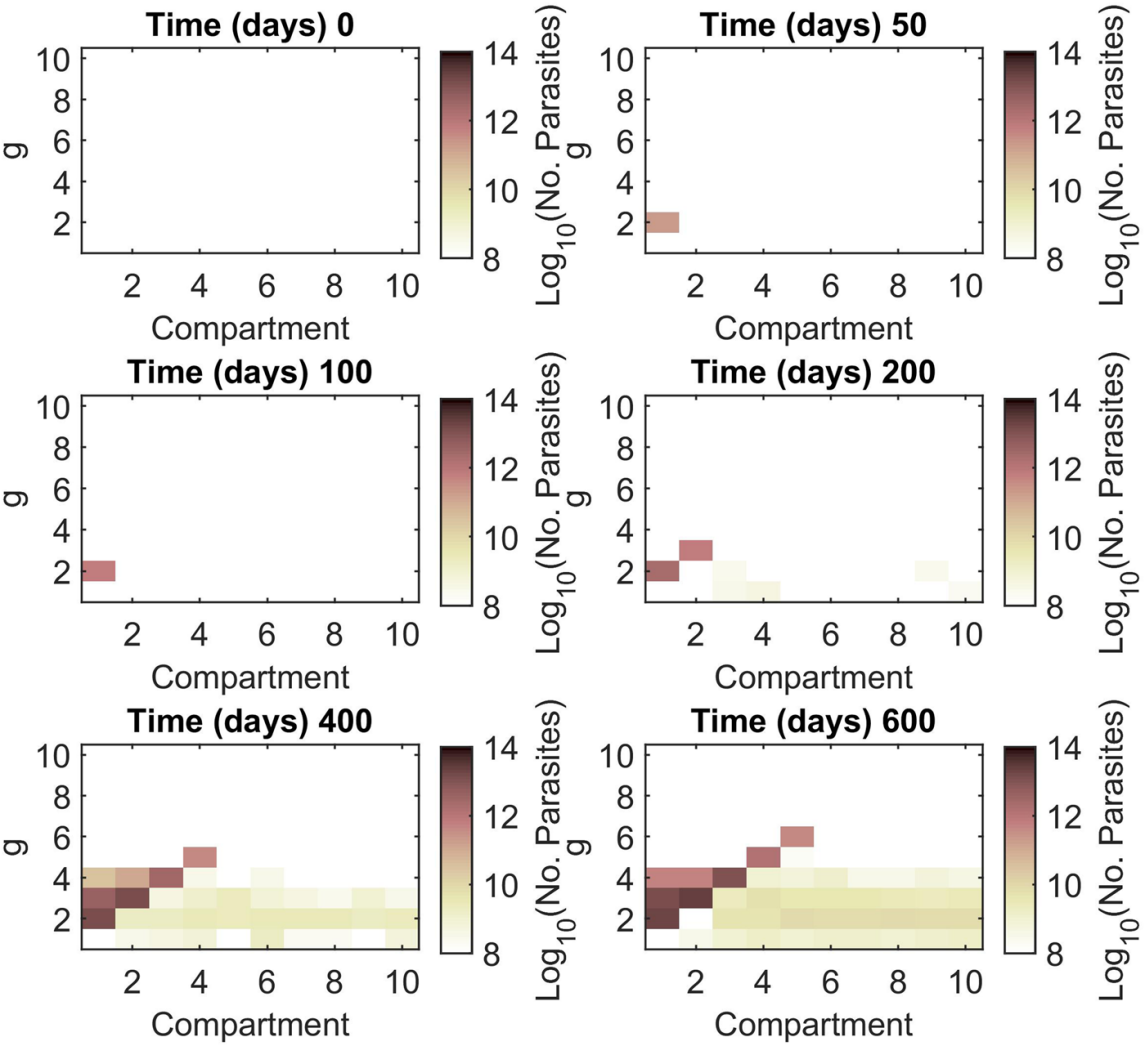
Snapshots showing the parasite population evolving antimalarial drug resistance. The colour maps indicate the number of infected red blood cells (IRBCs) of human individuals. Initially, 100 non-resistant parasites (*g* = 1) infect susceptible human individuals in compartment 1. The parasite population adapts and expands simultaneously to invade the other compartments. While most of the parasite population climbs the staircase to obtain the higher level of drug resistance, some of the low resistant population spreads horizontally to the areas with higher drug concentrations. Parameters: *μ*_*f*_ = 10^−7^ *day*^−1^, *μ*_*b*_ = 10^−4^ *day*^−1^, PRR = 10^3^, *and τ* = 0.1 *day*; all of the others are listed in Tables 1 and 2.

In this section, we investigate the evolutionary dynamics of antimalarial drug resistance in both heterogeneous and homogeneous environments. Figure 4 shows snapshots of the parasite population evolving antimalarial drug resistance (see also Supplementary Movie S1). The simulation started with 10 infected human hosts in compartment 1; each is infected with 10 non-resistant sporozoites (*g* = 1). These parasites reproduce in the infected hosts according to the proposed within-host parasite dynamics (Fig. 1A). The first resistant strain (*g* = 2) rises and dominates in compartment 1 at approximately day 39. At day 138, the parasites with *g* = 3 dominate in compartment 2 while the parasites with *g* = 1 can be found at some distant compartments (for example, compartments 5 and 7). At day 348, the parasites with *g* = 4 can be found. The parasites with *g* = 1 and 2 now spread to all of the compartments. As shown in Fig. 4, the parasite population grows and then spreads upward and rightward. Antimalarial drug resistance develops at the edges of the staircase and then spreads horizontally to the right compartments. At the end of the simulation, the coexistence of several parasite strains can be found in all of the compartments.

Importantly, the evolutionary dynamics exhibited in the heterogenous environment (Fig. 4) differ from those in the homogeneous environment. In the homogeneous environment (see Figs. S3–S6 in the supplementary results), resistance to antimalarial drugs occurs by chance. Although the resistance to low drug concentrations is easy to find, the resistance to higher drug concentrations is rare. By assuming that the parasites evolve drug resistance via a forward mutation that occurs only once in a generation, wild-type parasites cannot immediately resist high drug concentrations in only one mutation. In a homogeneous environment with a level-2 drug concentration (Fig. S5), the parasites require two forward mutations to survive. The time to find the parasite with *g* = 3 is approximately 765 days, while it takes only 140 days to find the parasite with *g* = 3 in the heterogeneous environment. In the homogeneous environment, two consecutive forward mutations are required to increase *g* to *g* = 3 before the parasite is cleared out by the drug. In contrast, in the heterogeneous environment, two forward mutations are not required to occur consecutively. Since the antimalarial drug concentration is increased stepwise, the parasites can rest and accumulate mutations before they go to the other compartments with higher drug concentrations.

**Figure 5:**
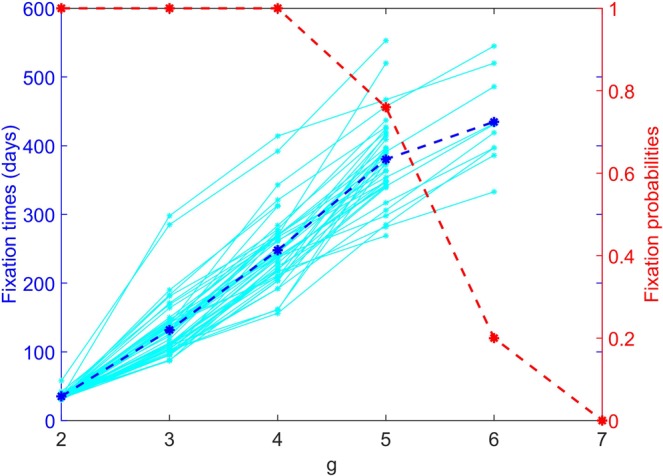
The fixation times and fixation probabilities of parasites. The left vertical axis shows the fixation times. Each cyan dashed line indicates the fixation time measured from the individual simulation. The blue solid line shows the mean fixation time averaged from 50 simulation runs. The right vertical axis shows the fixation probability. The red line shows the fixation probability calculated from 50 simulation runs.

Based on the proposed evolutionary model, under antimalarial treatment, only the parasites with *g > i* can survive in compartment *i*. To quantify the speed of the parasite adaptation, we therefore measured a fixation time of parasites with genotype *g*, which is defined as the earliest time that the parasites with *g* = *i* + 1 dominate in compartment *i*. Nevertheless, the fixation time will indicate only how fast the evolution of resistance is, but not how likely it will occur. Therefore, we also measured a fixation probability of the parasites with *g* = *i* + 1 in compartment *i* calculated from the number of simulation runs that the fixation of the parasite with *g* = *i* + 1 was found within 1,000 days since the treatment began divided by the total number of simulation runs. Figure 5 shows the measured fixation times and the corresponding fixation probabilities. Obviously, parasites with a lower resistant level can be found more easily than the higher resistant parasites.

### Sensitivity analysis

**Figure 6:**
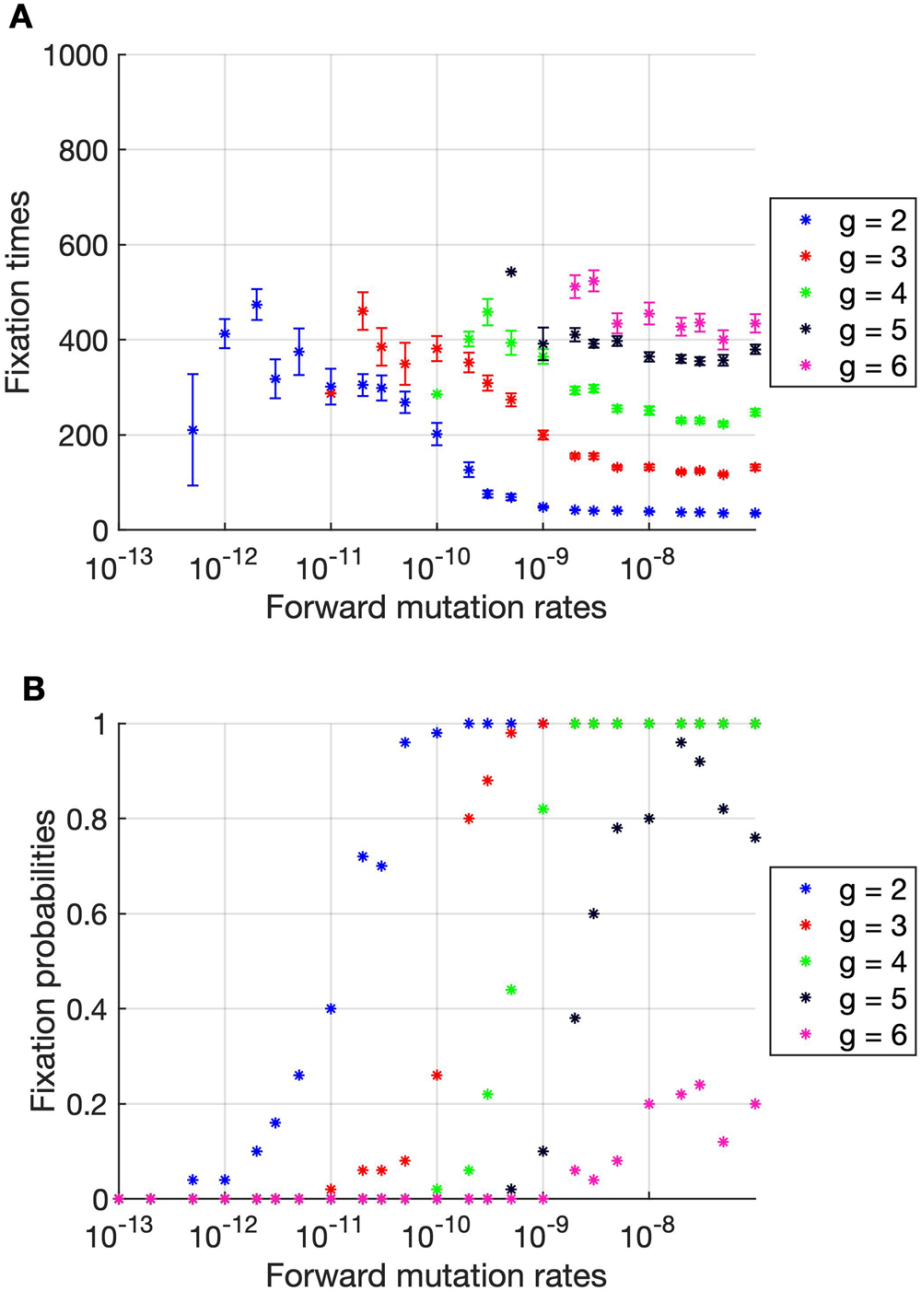
The fixation times and fixation probabilities as a function of the forward mutation rates. (**A**) The fixation times generally decrease when the forward mutation rates increase. The error bars show the standard error of the mean. (**B**) The fixation probabilities generally increase with the increasing forward mutation rates. The drug resistant parasites can be found more frequently when the forward mutation rate increases. The results were averaged from 50 simulations.

**Figure 7:**
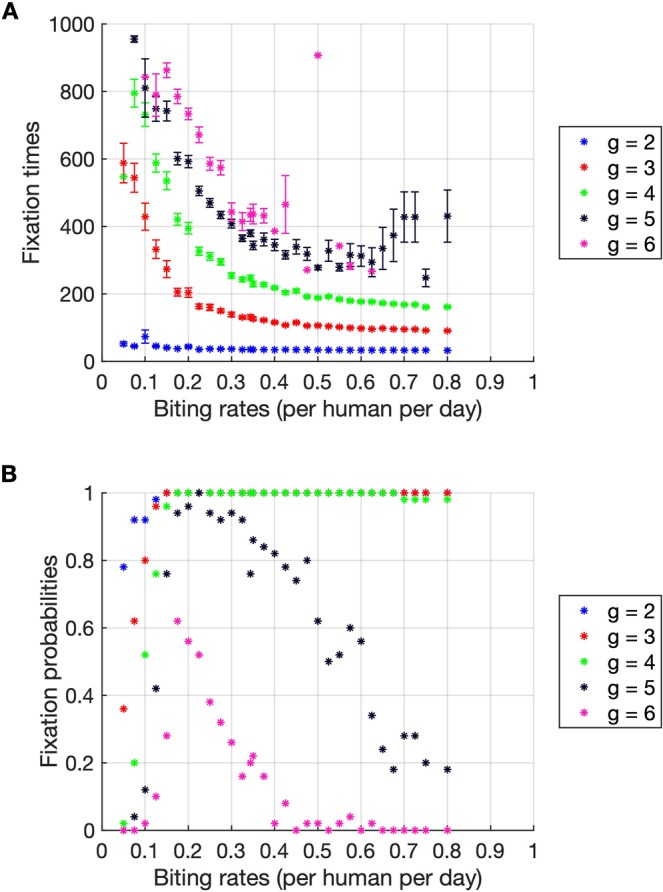
The fixation times and fixation probabilities as a function of the biting rates. (**A**) The fixation times decrease when the biting rates increase. If humans are bitten more frequently, drug resistant parasites will emerge earlier. The error bars show the standard error of the mean. (**B**) In the regime of a high biting rate, the fixation probabilities of parasites with higher *g* decrease with the increasing biting rates. The results were averaged from 50 simulations.

In this section, a sensitivity analysis of certain model parameters on antimalarial drug resistance evolution is performed. We first investigated the effects of the parasite mutation rates on the parasite evolutionary dynamics. Obviously, the resistant parasites usually emerge faster and more frequently when the forward mutation rate increases (Fig. 6). Next, a sensitivity analysis of the biting rate was performed. This parameter can affect the speed of parasite migration. If humans are bitten more frequently, the parasites are more likely to be transmitted between human individuals. As shown in Fig. 7, the relationship between the fixation time, the fixation probability, and the biting rate may be classified into two scenarios. In the low biting rate scenario (less than 0.3 per human per day), the fixation times of drug resistant parasites decrease rapidly as the biting rates increase. After the biting rate exceeds 0.3 per human per day (the high transmission scenario), the fixation times continue to decrease but at slower rates. More interestingly, in the low transmission scenario, the fixation probabilities of resistant parasites generally increase as the biting rates increase. In contrast, in the high transmission scenario, the fixation probabilities of higher resistant parasites, for example, *g* = 5 and 6, decrease as the biting rates increase. This may be because, in the high transmission scenario, the parasites spread rapidly, and therefore the strains with lower resistant level can occupy most compartments before the parasites with higher resistant level arrive. Next, we investigated the effects of the parasite reduction ratios (PRRs) on the parasite evolutionary dynamics. As shown in Fig. S7A in the supplementary information section, the fixation times of the resistant parasites do not depend on the PRRs. This is similar to the result found in the study of antibiotic resistance evolution in bacteria, in which the bacterial adaptation rate does not depend on the drug killing rate^13^. In addition, we found that the fixation probabilities of higher resistant parasites (*g* = 5 and 6) slightly decreased with the increasing PRRs (Fig. S7B). This indicates that higher resistant parasites might be less likely to be found if they are under antimalarial treatment with a higher PRR. Finally, we investigated whether limiting the maximum number of gametocytes that a mosquito can take during a blood meal to 20 will affect the evolutionary dynamics of parasites or not. As shown in Fig. S8, we found that the fixation times and the fixation probabilities of the resistant parasites do not significantly depend on the maximum number of gametocytes that a mosquito can take during a blood meal.

Similar to the antibiotic resistance evolution in bacteria, the adaptation of antimalarial resistant parasites usually carries a fitness cost of mutation^37–39^. To investigate the effect of the fitness cost of mutation on the fixation time and the fixation probability, we slightly modified the within-host model of malaria parasites. Following the methodology presented in^13^, we assumed that all parasites in mosquitoes and humans, except only the blood-stage merozoites, grow a factor 1 – *s* more slowly than the drug-sensitive parasites, where *s* is a normalised fitness cost of mutation. In our model, only the blood-stage merozoites are under the drug pressure; they, therefore, gain an advantage from being resistant. The fitness gain was assumed to be equal to the fitness cost of mutation so that there is no fitness cost for the blood-stage merozoites. However, if the blood-stage merozoites are more resistant than necessary (*g* > *i*), their fitness cost will be dominated; they, therefore, will grow a factor 1 – *s* more slowly than the drug-sensitive merozoites. As shown in Fig. S9, we found that the fitness cost of mutation can prolong the fixation time of the resistant parasites excepts the resistant parasites with *g* = 2 where only one step of mutation is required. In addition, the fixation probability of high resistant parasites (*g* = 3 − 6) also decreases when the fitness cost of mutation increases.

## Discussion

The staircase model proposed in^40^ provides a basic framework for studying the roles of a concentration gradient in drug resistance evolution. The original staircase model was used to assess the evolutionary dynamics of antibiotic resistance in bacteria. In the present report, we adapted the original framework to investigate the roles of a concentration gradient in malaria drug resistance evolution in malaria parasites. Since the life cycle of malaria parasites is much more complicated than that of bacteria, studying the roles of a concentration gradient in the malaria drug resistance evolution process in parasites is challenging.

We proposed a novel stochastic model to investigate the roles of a concentration gradient on malaria drug resistance evolution. The aims of the model were to investigate the evolutionary dynamics of malaria drug resistance within the human hosts and the spread of resistant parasites in heterogeneous environments. Our model consists of many sub-compartments representing individual hosts and mosquitoes. Parasites can cross to the other compartments only via human travel and mosquito bites. Since a malaria parasite develops through several life stages and lives within both the human host and mosquito hosts, malaria treatment will not affect all parasite stages. Parasites in certain stages will never encounter the antimalarial drugs, for example, all stages in the mosquito. Environments in which the parasites can live without being killed by an antimalarial drug act as sanctuaries for the parasites to avoid the antimalarial drug. This is different from the original staircase model in which all of the bacteria in the same compartment encounter the same drug concentration^40^.

Our work focuses on the evolutionary dynamics of malaria drug resistance within a human body and the transmission of the resistant parasites among human and mosquito populations. To investigate the evolutionary dynamics of malaria drug resistance on the scale of the parasite’s population in a human body, the within-host population dynamics model was constructed to simulate the whole life cycle of *P. falciparum*^22,24–30,33,36^. All of the model parameters were based on experimental and clinical reports (see Table 1). Although parasites can independently live within a single human host, they can be transmitted to other human individuals via mosquito bites. In addition, as the parasites live within individual hosts, they can travel to different geographical locations with their hosts. To take into account the effect of human mobility in carrying the parasites to different geographical locations, the between-host transmission dynamics are also integrated into the model. Moreover, the notable roles of spatial heterogeneity were investigated by comparing the fixation times of parasites in homogeneous environments with those in heterogeneous environments. To the best of our knowledge, this study presents the first computational analysis of the roles of a concentration gradient on the evolutionary dynamics of malaria drug resistance.

The within-host population dynamics model was used to simulate the parasite population dynamics within both individual humans and mosquitos (Fig. 1A,B). The model was constructed based on the experimental and reported clinical data^22,24–30^. The simulation results were also validated with the clinical data^33,36^. The artemisinin treatment was employed in our model. Artemisinin exerts its malaria treatment effect by targeting the blood stage parasites^6^. Artemisinin is one of the most effective drugs and can be combined with other compounds for combination therapy. We found that the simulation results are in good agreement with the experimental data (Fig. 3)^35^.

The within-host population dynamics model was then extended to include the between-host transmission dynamics (Fig. 1C). The mosquito infection probability, which is the probability that a mosquito will carry the parasites with a blood meal from a target human, is determined by the gametocyte density within the target host (Fig. S1)^31^. Furthermore, to integrate the effects of spatial heterogeneity, both human and mosquito populations were divided into subpopulations living in different spatial compartments. Each compartment represents a co-living area of human and mosquito hosts (Fig. S2). By assuming that humans can travel to the other compartments, the parasites can migrate to the other compartments along with the human hosts. To describe the movement of human individuals, the human mobility model was also integrated into the transmission dynamics^32^.

In heterogeneous environments, a concentration gradient exists across the environment. Similar to the result found in the study of antibiotic resistance evolution, the antimalarial drug gradient can lead to a mode of adaptation that is different from those occurring in homogeneous environments. Our results show that the time to find high-resistant strains in heterogeneous environments is shorter than that in homogeneous environments (Figs. 4 and S3–S6). In heterogeneous environments, the sources and sinks of parasites are provided by the concentration gradient. We found that in heterogeneous environments, the first drug resistant parasite appears at approximately day 36, which is close to the time to find the first resistant parasite in the homogeneous environment with the level-1 drug concentration (Fig. S4). In this case, the parasites need only one forward mutation to increase *g* from *g* = 1 to *g* = 2 to resist the antimalarial drug. However, in the homogeneous environment with a higher level of drug concentration, the evolution of the antimalarial drug resistance is significantly slower than that in the heterogeneous environment (Figs. S5 and S6). In this case, two or more consecutive steps of the forward mutation are required for the parasites to survive in the environment.

We found that the rate of resistance evolution in the high transmission setting (high mosquito biting rate) is slower than in the low transmission setting. This is because in the high transmission setting, the lower resistance strains have a higher chance to migrate to and fully occupy the distant compartments before the strains with higher resistance level emerge. As parasites can linger within recovered individuals who show no signs of symptoms and will not seek medical treatment, the parasites can survive and spread to the distant compartments without being killed by antimalarial drugs.

Similar to the antibiotic resistance evolution in bacteria^13^, we found that the killing rates of antimalarial drugs are not likely to affect the evolutionary rate of antimalarial drug resistance (Fig. S7). This is probably because, under the concentration gradient, the resistance evolution can most likely occur through two “evolutionary paths”^13^. Each path involves only one mutation and one migration event. In the first path, the parasite first mutates in a compartment with a lower drug concentration and then migrates to another compartment with a higher drug concentration. On the other hand, in the second path, the order of mutation and migration is opposite. The migration to a compartment with a higher drug concentration occurs first and then is followed by a mutation. Increasing the PRR decreases the time that the parasites with lower resistant level can live under the staircase and therefore only affects the second path^13^. When the drug killing rate is lower than the parasite reproduction rate, the resistance evolution through the two paths is likely to occur at the same rate. However, when the antimalarial drug killing rate is higher than the parasite reproduction rate, the rate of the resistance evolution through the second path decreases since lower resistant parasites are killed more rapidly under the staircase. In this case, the resistance evolution through the first path is dominant and is not affected by the increase in the PRR.

It is worth noting that, to simplify the within-host model, we assume that gametocytes can be produced immediately after the production of merozoites in the liver. This assumption might not be realistic since literature suggests that the liver stage of infection should last around a week before the liver merozoites are released into the host circulation, where they can invade red blood cells and subsequently produce gametocytes^41,42^. However, we do believe that the conclusion of this work is not affected by this assumption. This is because the number of merozoites produced during the liver stage is tiny as compared to the number of merozoites that can be produced in the blood stage (about 10^5^ merozoites can be produced in the liver stage while 10^12^ merozoites can be produced in the blood stage). Therefore, the number of gametocytes that may be produced during the liver stage is very small as compared to the number of gametocytes produced from the blood merozoites. We have confirmed this by running a slightly modified version of our within-host model in which the rate (*r*) that the liver merozoites are released to the bloodstream and invade the red blood cells was changed to:

3 $$r=\{\begin{array}{cc}\,0, & t\le {t}_{0}\\ 1\,-\,N/K, & N < K,\,{\rm{and}}\,t > {t}_{0}\\ 0,\, & N\ge K,\,{\rm{and}}\,t > {t}_{0}\end{array}$$

In this modified model, the liver merozoites cannot infect the red blood cells and, therefore, cannot produce gametocytes before a time threshold *t*_0_. We ran the modified model with different values of *t*_0_ and calculated the mosquito infection probability using the relationship shown in Fig. S1A. As shown in Fig. S1B in the Supplementary Material, we found that restricting the gametocytes to be produced precisely after 1–10 days does not significantly affect the mosquito infection probability. The effect of *t*_0_ on the infection probability is noticeable only when *t*_0_ is extreme such as 12–13 days, which might be rare in reality^20,43^. Note also that even the case of *t*_0_ = 1 day, our calculated mosquito infection probability starts to increase only after approximately 10 days; this agrees well with the fact that malaria has an incubation period of about 6–14 days in human^44^.

We presented a stochastic combined within- and between-hosts evolutionary dynamics model of malaria parasites to investigate the antimalarial resistance evolution in the presence of an antimalarial concentration gradient. Although the proposed evolutionary model was build based mainly on available experimental and clinical data related to antimalarial resistance, the antimalarial concentration gradient employed in this study was highly idealised. The pattern of the concentration gradient was purposefully kept simple so that the roles of the concentration gradient could be quantitatively characterized. In addition, to allow a direct comparison of the roles of a concentration gradient in antimalarial resistance evolution to those in the antibiotic resistance evolution, a linear concentration gradient was employed in this study; as this kind of concentration gradient was used in previous studies of antibiotic resistance evolution^13,45^. However, we do expect that several basic results found in this study will hold up for a more realistic concentration gradient as long as there exist compartments with a low antimalarial concentration where they can act as sanctuaries (or reservoirs) and allow mutations to be accumulated.

## Supplementary Information

Supplementary Information 1.

Supplementary Information 2.

## Data Availability

The authors declare that the data supporting the findings of this study are available within the paper and its Supplementary Information file. Computer code are available from the authors upon reasonable request.
