# Supplementary material for: Role of a Concentration Gradient in Malaria Drug Resistance Evolution: A Combined within- and between-Hosts Modelling Approach
Source: Sci Rep. 2020 Apr 10;10:6219. doi: 10.1038/s41598-020-63283-2 (PMC7148383; doi:10.1038/s41598-020-63283-2)
Supplement: Supplementary file 1 — Supplementary Information 1. [file 41598_2020_63283_MOESM1_ESM.pdf]

Supplementary Information:

Role of a Concentration Gradient in Malaria Drug Resistance Evolution: A Combined within- and between-Hosts Modelling Approach

Suwat Romphosri,[1] Suttikiat Changruenngam,[1] Thanat Chookajorn,[2] Charin Modchang[1,3,4,*]

[1]Biophysics Group, Department of Physics, Faculty of Science, Mahidol University, Bangkok 10400, Thailand

[2]Genomics and Evolutionary Medicine Unit (GEM), Center of Excellence in Malaria Research, Faculty of Tropical Medicine, Mahidol University, Bangkok, Thailand

[3]Centre of Excellence in Mathematics, CHE, Bangkok 10400, Thailand

[4]Thailand Center of Excellence in Physics, CHE, 328 Si Ayutthaya Road, Bangkok 10400, Thailand

[*]Corresponding author email: charin.mod@mahidol.edu

Supplementary methods

The between-host transmission dynamics model

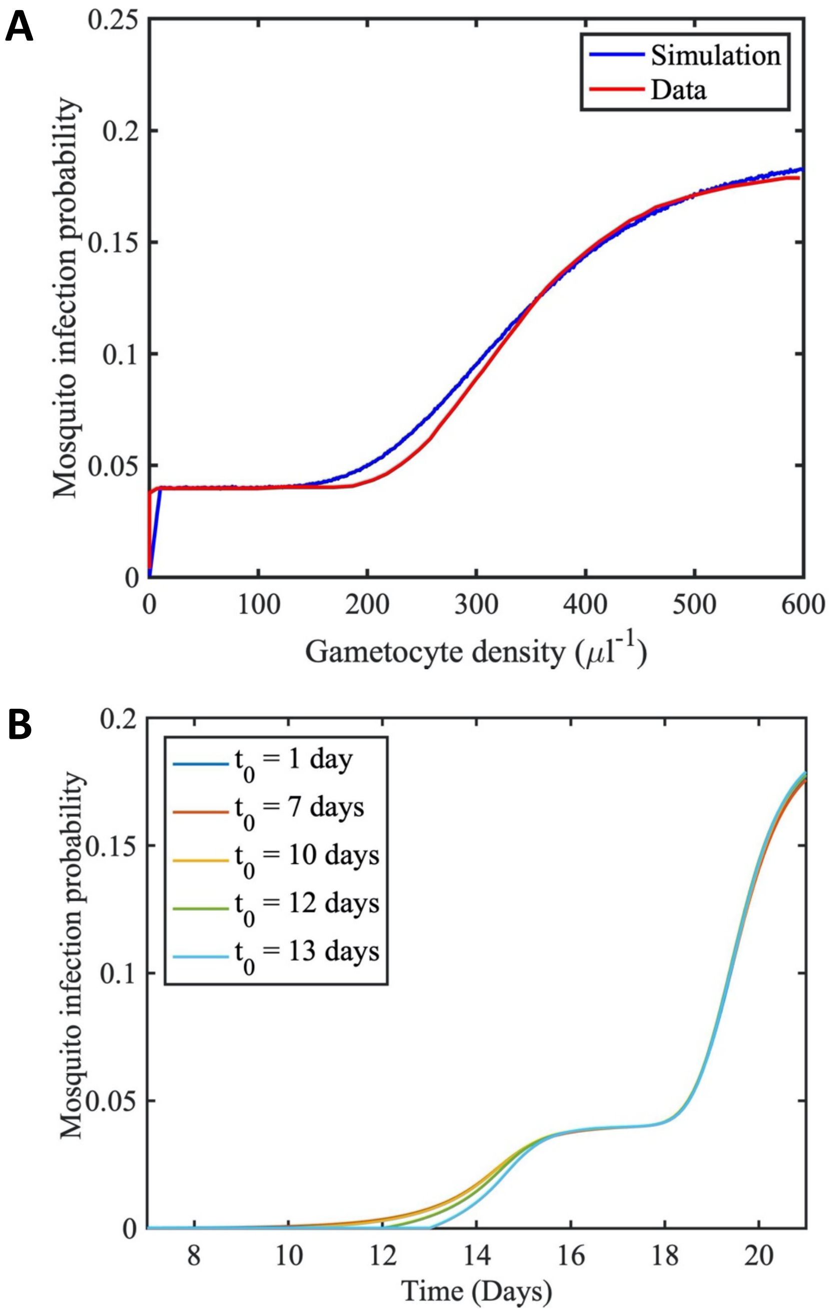

Figure S1. The mosquito infection probability. (A) The relationship between the mosquito infection probability and the gametocyte density. The red line shows the mean of the observed data adapted from [1]. The blue line shows the simulated result. The simulation results were obtained by simulating the relationship shown in Eq. S1. **(B)** The mosquito infection probability as a function of time in the case of no antimalarial treatment in human. The probability was measured by running the modified model with a different value of the time

threshold (t_0) (see Eq. 3 in the main text). Note that the curves for $t_0 = 1$, 7, and 10 days overlap entirely.

Figure S1 shows the relationship between the mosquito infection probability and the gametocyte density. The experimental data presented in this figure were adapted from reference [1]. The relationship is divided into two regimes. In a low gametocyte density regime, the probability of mosquito infection is very sensitive to the change in the gametocyte density. It rapidly increases from 0 to 0.04 when the gametocyte density increases from 0 to 10 gametocytes per μl. In our model, a linear relationship between the infection probability and the gametocyte density is assumed (**Figure S1**, inset). In contrast, in a high gametocyte density regime, the infection probability increases more slowly with the gametocyte density and approaches the maximum value at 0.18. In this regime, the relationship between the mosquito infection probability and the gametocyte density is modelled using the Gompertz equation [1]. The following equation summarises the relationship of the mosquito infection probability (P_{Inf}) and the gametocyte density:

$$P_{Inf}\,(n_{ih}) = \begin{cases} \frac{\alpha}{10} n, & 0 < n \le 10 \\ \alpha + \beta \exp(\gamma \exp(-\delta n)), & n > 10 \end{cases}, \qquad \text{(S1)}$$

where n is the density of gametocytes (gametocytes per μl) within the human host, $\alpha = 0.04, \beta = 0.15, \gamma = 0.2$, and $\delta = 0.01$.

The spatial heterogeneity and evolutionary dynamics

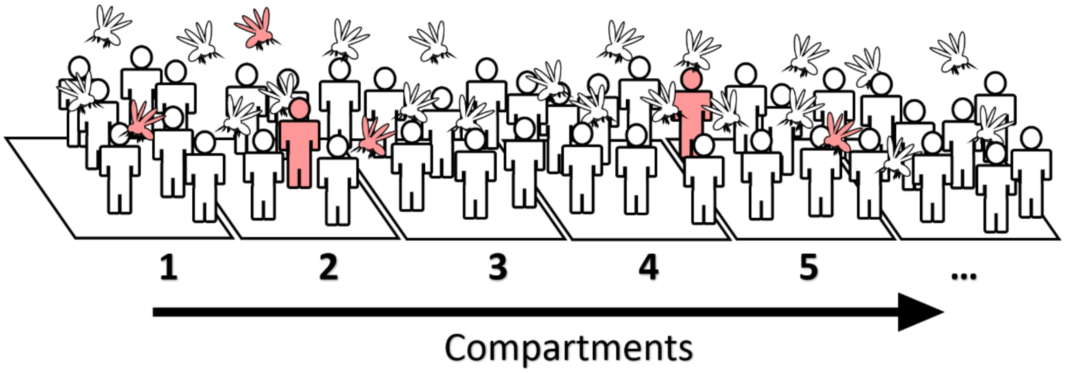

Figure S2. The spatial compartments co-living by humans and mosquitos. The compartments are arranged in one-dimensional space. Each compartment has a width of 1 km.

Supplementary results

The evolutionary dynamics of antimalarial drug resistance in homogeneous environments

To observe the effects of a drug concentration gradient, the evolutionary dynamics in homogeneous environments were also investigated. Three homogeneous drug concentration patterns were employed in this study (**Figure S3**).

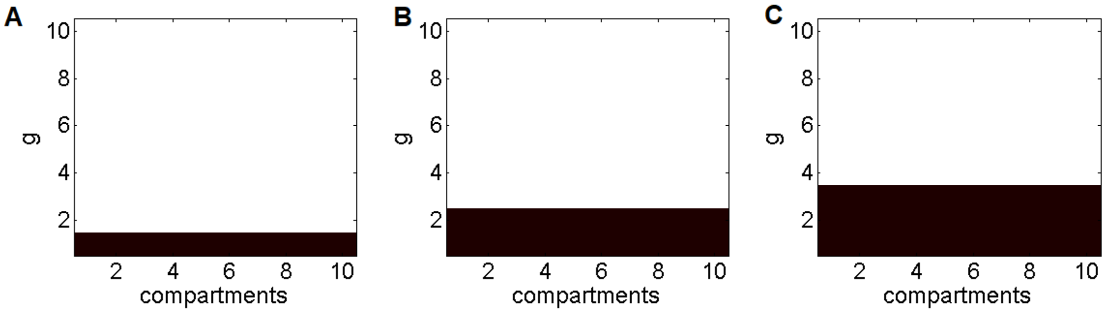

Figure S3. Homogeneous environments. Three homogeneous environments with different levels of uniform drug concentration. **(A)** level-1, **(B)** level-2, and **(C)** level-3.

In the homogeneous environment with the level-1 antimalarial drug concentration (**Figure S4**), only a single step of the forward mutation is required to make parasites survive in the environment. The first drug resistant parasite appears at day 36 and then it multiplies and spreads throughout the environment. At day 317, only the parasites with $g = 2$ remain in the environment. Because the level-1 concentration cannot kill parasites with $g = 2$, the population increases quickly while the non-resistant population is wiped out by the drug. As the gametocyte transmission probability depends on the number of available gametocytes, the resistant strains have a higher chance of spreading than the non-resistant strains. At the end of the simulation, only the parasites with $g = 2$ remain in the environment.

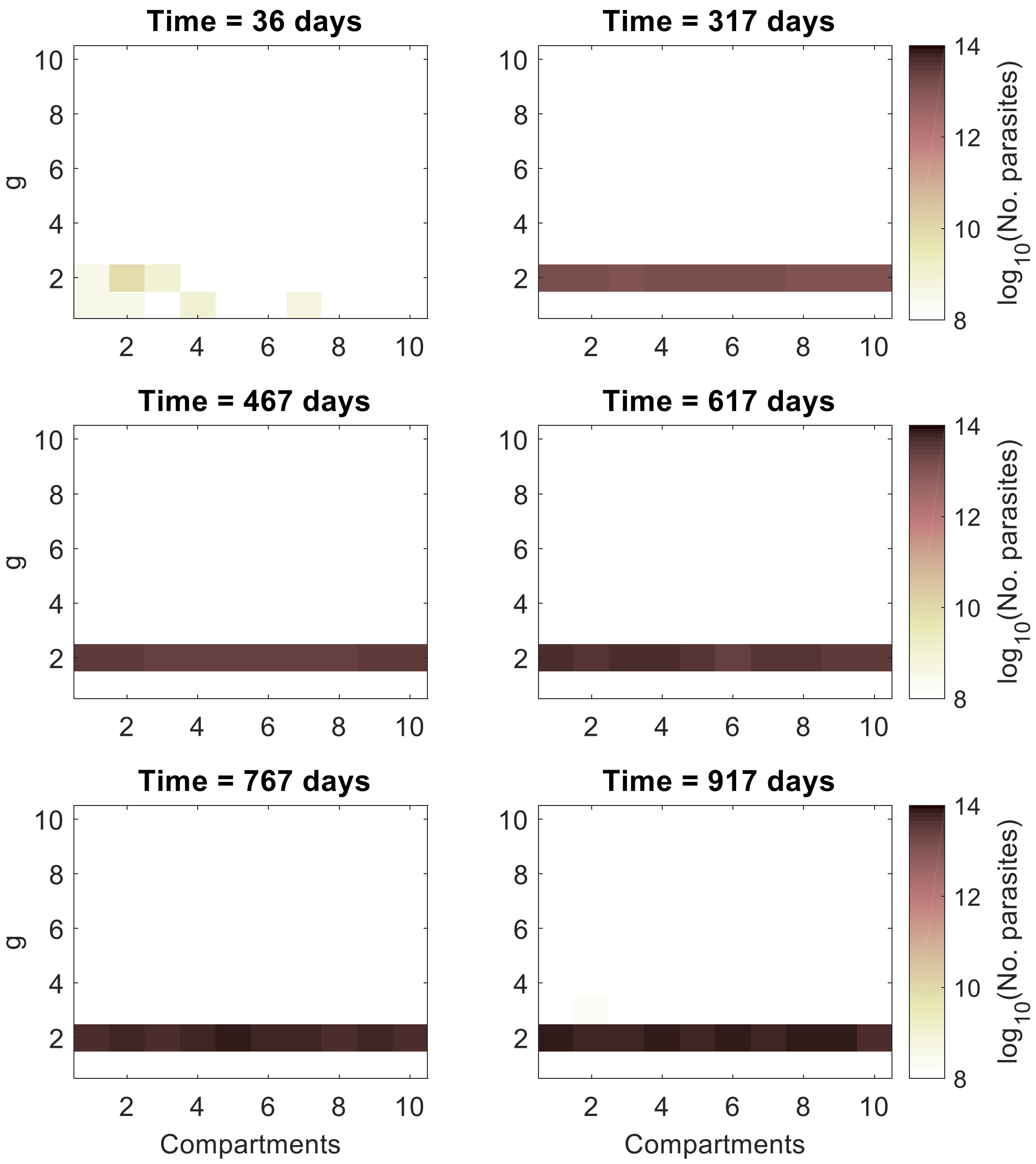

Figure S4. Antimalarial drug resistance evolution in the homogeneous environment with level-1 drug concentration. Snapshots of the parasite population evolving antimalarial drug resistance. The colourmaps indicate the number of blood stage parasites in humans. Initially, the parasites with $g = 1$ are injected into humans in compartment 1. The parasites with $g = 2$ appear and dominate in the environment at day 36. The resistant parasites live and grow freely, while the non-resistant parasites are cleared out by the drug. Parameters: $\mu_f = 10^{-7}$ day^{-1}, $\mu_b = 10^{-4}$ day^{-1}, PRR $= 10^3$, and $\tau = 0.1$ day.

In the homogeneous environment with level-2 antimalarial drug concentration (**Figure S5**), the antimalarial drug concentration is higher than the previous case by one level. Parasites with $g \geq 3$ can survive in the environment while parasites with $g < 3$ will be cleared out by the drug. Comparing the heterogeneous and the level-1 homogeneous environment, the evolution of the antimalarial drug resistance in the level-2 drug concentration environment is slower. Two consecutive steps of the forward mutation are required to increase g to 3. After the parasites with $g = 1$ mutate to $g = 2$, another forward mutation is required before the mutated parasite is cleared out by the drug. In **Figure S5**, the parasites with $g = 3$ first appear at day 765, which is longer than the heterogeneous environment.

Finally, in the homogeneous environment with the level-3 antimalarial drug concentration (**Figure S6**), the drug resistant parasites are very rare. Only the parasites with $g \geq 4$ can survive in this environment. In 50 model simulations, we did not find any parasites with $g \geq 4$. However, even if the parasites cannot survive under the drug concentration, there is still a time gap for the parasites to transmit. This is because before the number of parasites in an infected human reaches 10^9, the drug treatment is not applied to the infected individuals. During this time, the parasites can be transmitted to other individuals. At the end of the simulation, only the non-resistant parasites are uniformly distributed in the environment.

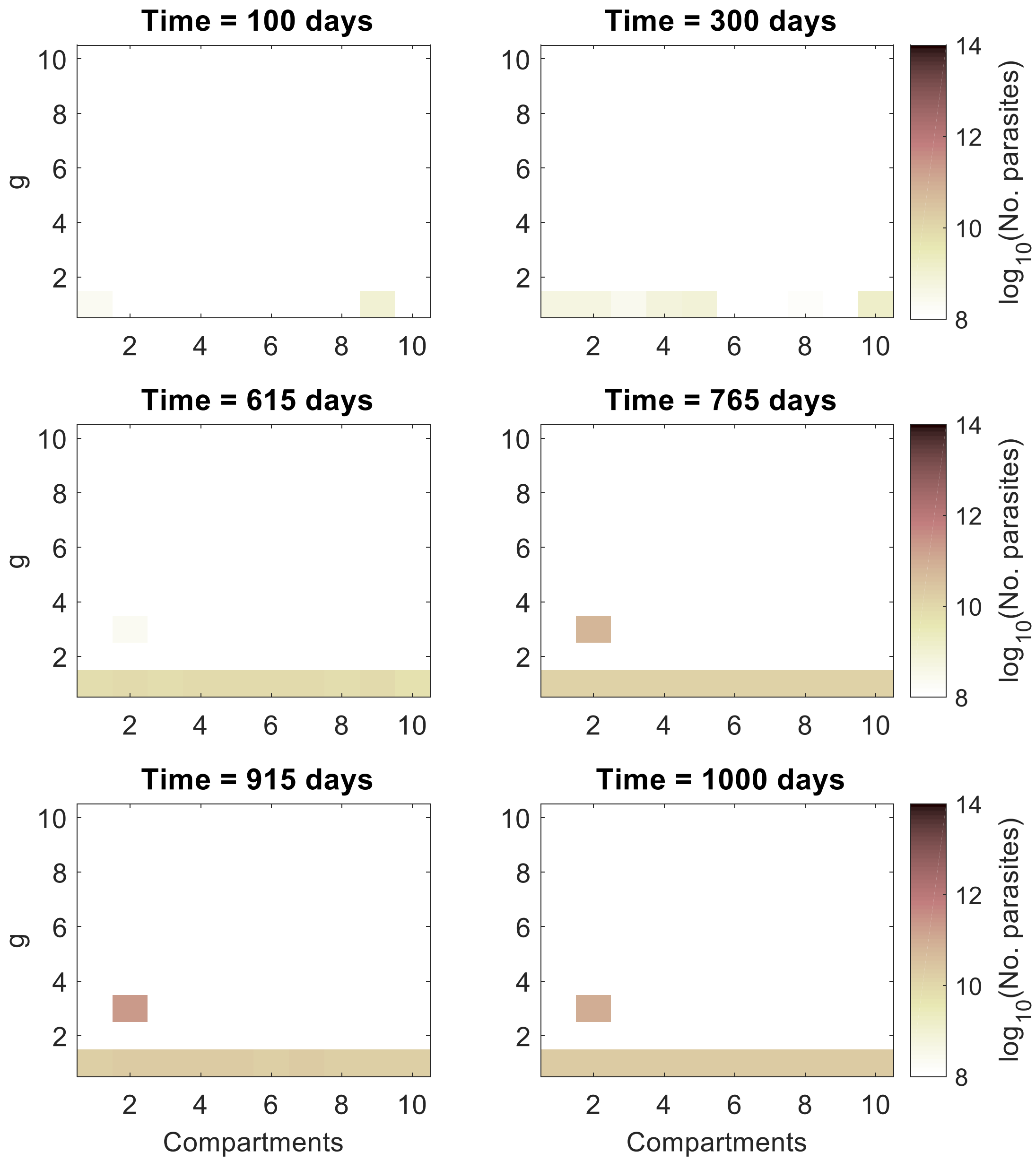

Figure S5. Antimalarial drug resistance evolution in the homogeneous environment with level-2 drug concentration. Snapshots of the parasite population evolving antimalarial drug resistance. The shaded areas indicate the number of blood stage parasites in humans. Initially, the parasites were non-resistant and incubating in compartment 1. The $g = 3$ parasites can be found at day 765, while parasites with $g = 2$ cannot be observed. Parameters: $\mu_f = 10^{-7}$ day^{-1}, $\mu_b = 10^{-4}$ day^{-1}, PRR $= 10^3$, and $\tau = 0.1$ day.

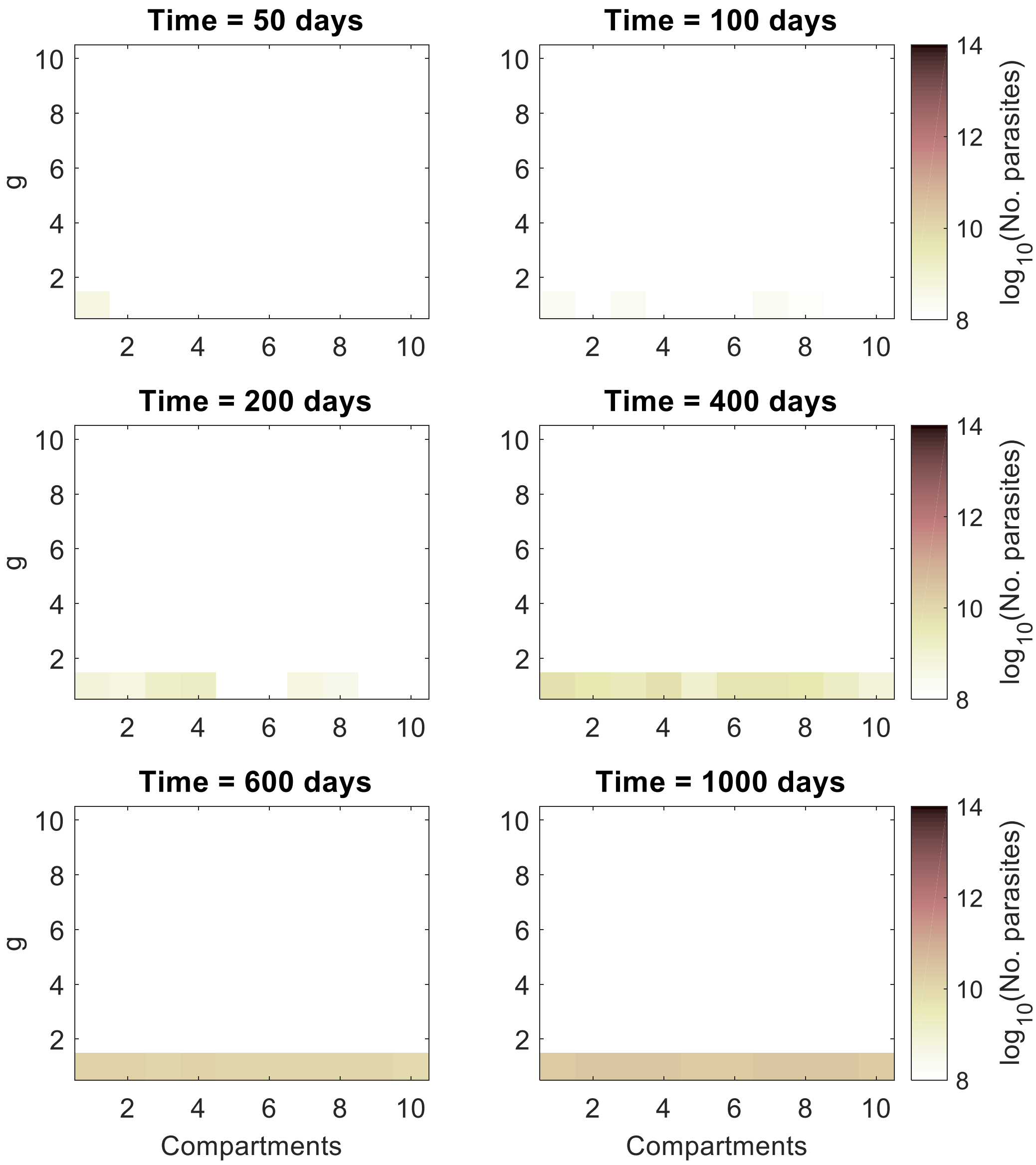

Figure S6. Antimalarial drug resistance evolution in the homogeneous environment with level-3 drug concentration. Snapshots of the parasite population evolving antimalarial drug resistance. The colourmaps indicate the number of blood stage parasites in humans. Initially, the parasites were non-resistant and incubating in compartment 1. Since the drug concentration is very high, the parasites need to mutate forward and consecutively three times to gain enough resistance, which is very rare. We did not observe any drug resistant parasite in 50 model simulations. Parameters: $\mu_f = 10^{-7}\ \text{day}^{-1}, \mu_b = 10^{-4}\ \text{day}^{-1}$, PRR $= 10^3$, and $\tau = 0.1$ day.

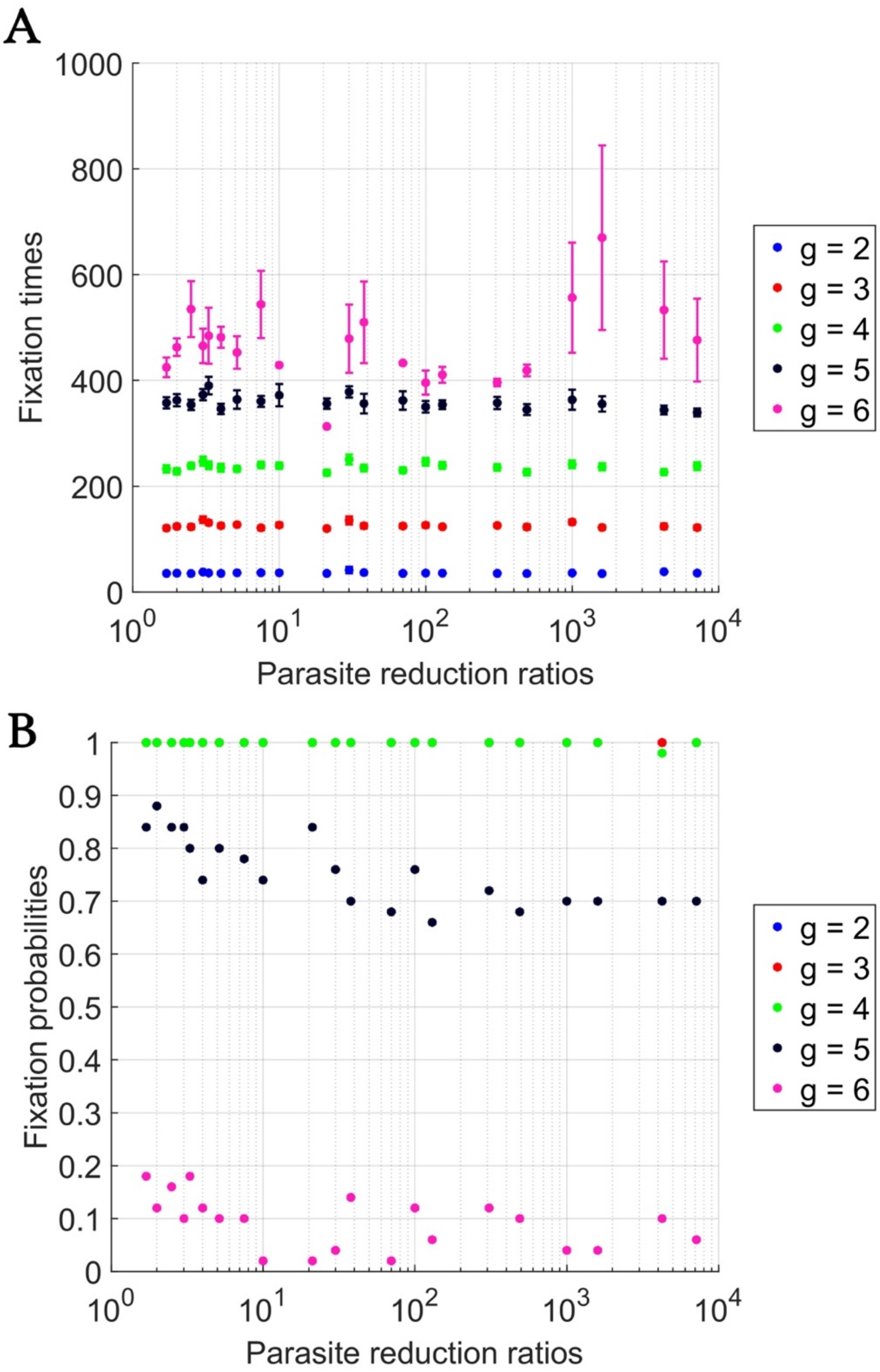

Figure S7. The fixation times and fixation probabilities as a function of the parasite reduction ratios. (A) The fixation time of parasites with the same genotype does not depend on the PRRs. The error bars show the standard error of the mean. **(B)** The fixation probabilities of the parasites. The results were averaged from 50 simulations.

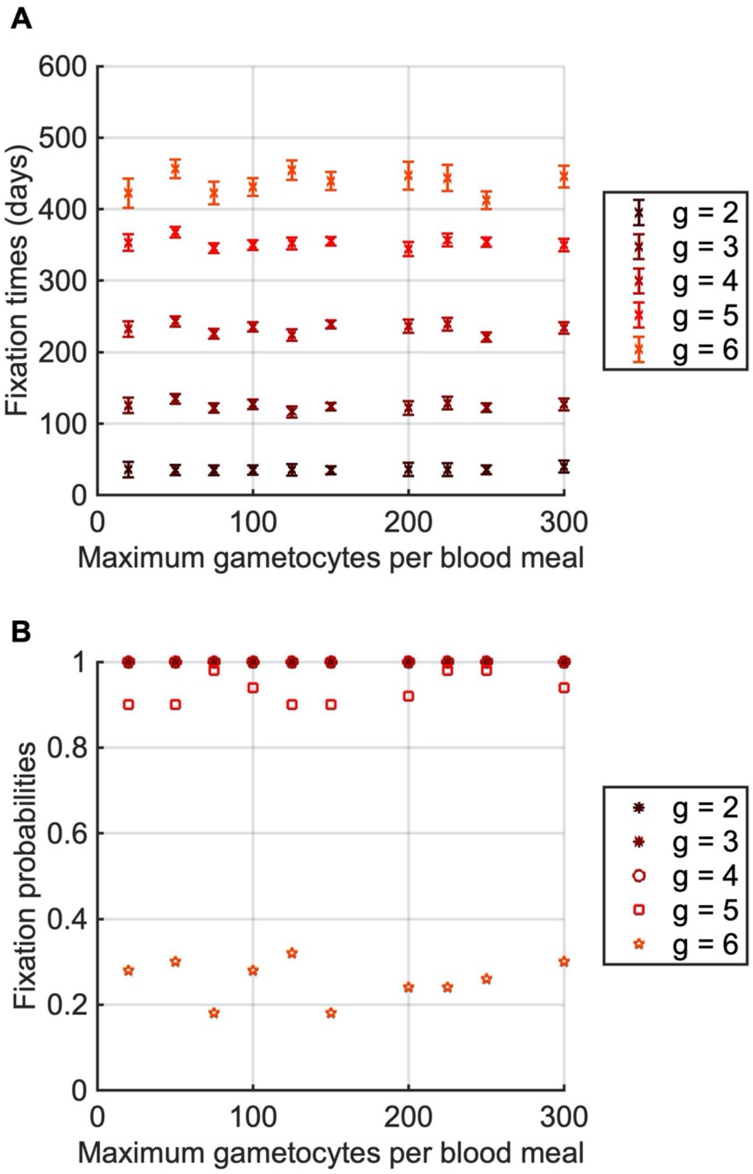

Figure S8. The fixation times and the fixation probabilities as a function of the maximum number of taken gametocytes per blood meal. (A) The fixation times of parasites with the same genotype does not significantly depend on the maximum number of gametocytes that are allowed to be taken during a blood meal. The error bars show the standard error of the mean. The results were averaged from 50 simulations. **(B)** The fixation probabilities of the parasites.

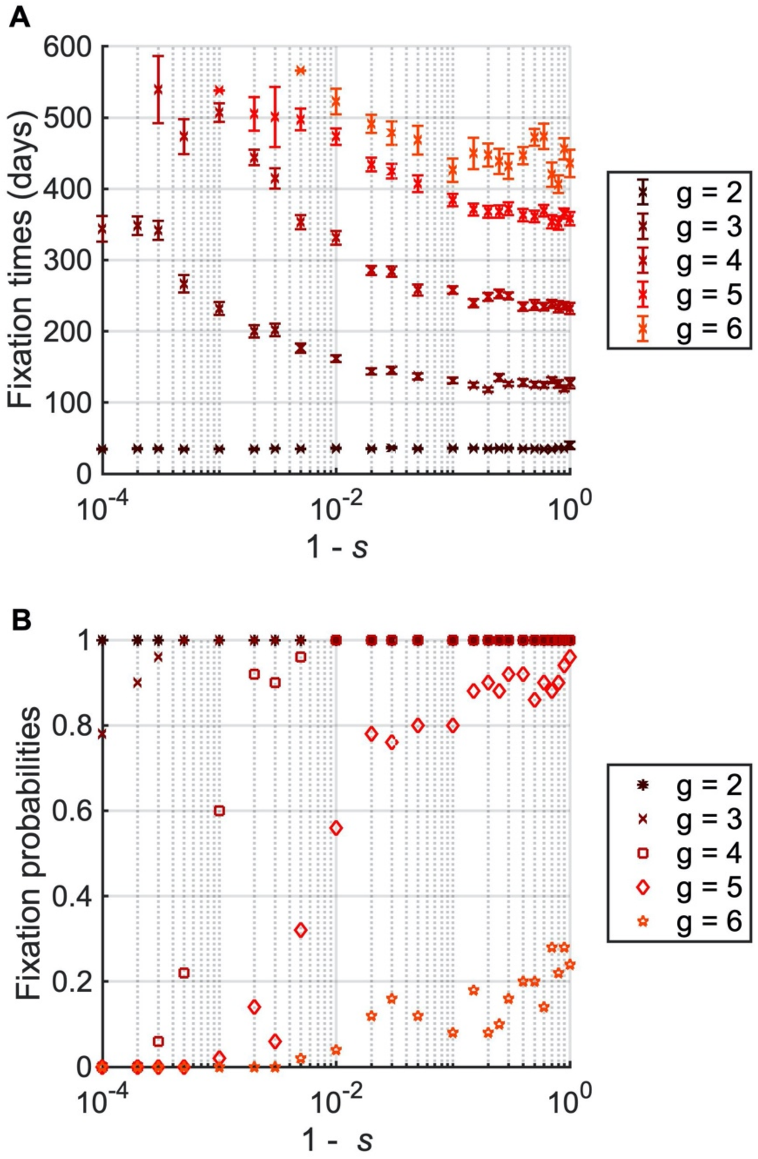

Figure S9. The fixation times and the fixation probabilities as a function of the fitness cost of mutation.

Reference:

1 Churcher, T. S. *et al.* Predicting mosquito infection from Plasmodium falciparum gametocyte density and estimating the reservoir of infection. *eLife* **2**, e00626, doi:10.7554/eLife.00626 (2013).
