# Supplementary figures and images for: Role of a Concentration Gradient in Malaria Drug Resistance Evolution: A Combined within- and between-Hosts Modelling Approach
Source: Sci Rep. 2020 Apr 10;10:6219. doi: 10.1038/s41598-020-63283-2 (PMC7148383; doi:10.1038/s41598-020-63283-2)

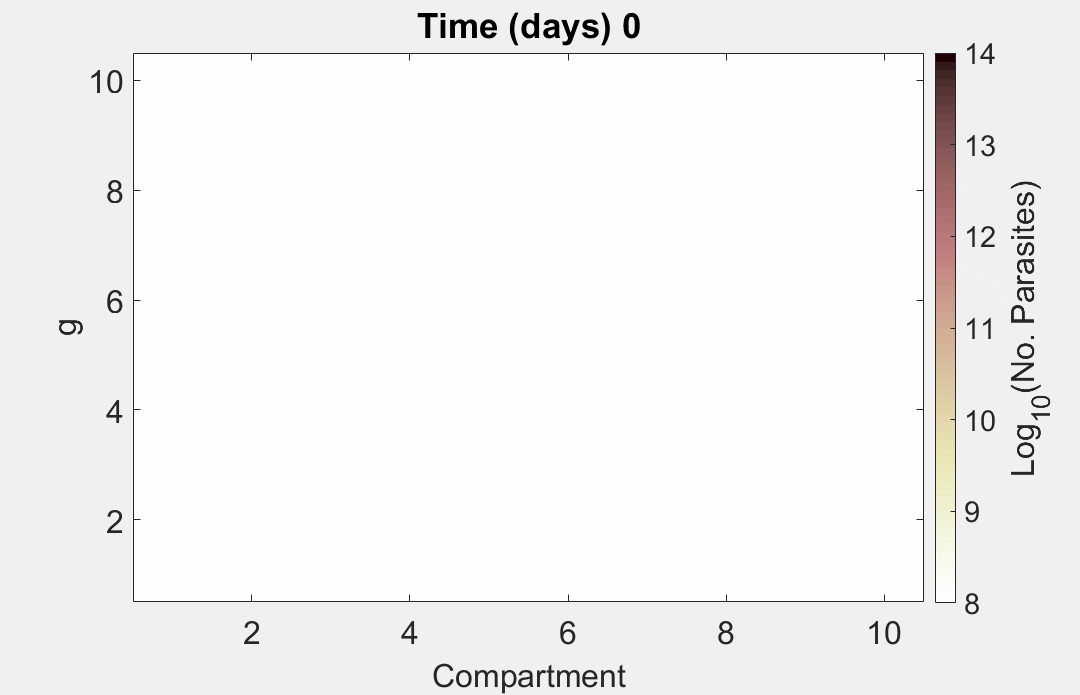

Supplement: Supplementary file 2 — Supplementary Information 2. [file 41598_2020_63283_MOESM2_ESM.gif]
